# Machine Learning to Predict Interim Response in Pediatric Classical Hodgkin Lymphoma Using Affordable Blood Tests

**DOI:** 10.1200/GO.23.00435

**Published:** 2024-10-24

**Authors:** Jennifer A. Geel, Artsiom Hramyka, Jan du Plessis, Yasmin Goga, Anel Van Zyl, Marc G. Hendricks, Thanushree Naidoo, Rema Mathew, Lizette Louw, Amy Carr, Beverley Neethling, Tanya M. Schickerling, Fareed Omar, Liezl Du Plessis, Elelwani Madzhia, Vhutshilo Netshituni, Katherine Eyal, Thandeka V.Z. Ngcana, Tom Kelsey, Daynia E. Ballott, Monika L. Metzger, South African Children's Cancer Study Group

**Affiliations:** ^1^ Department of Paediatrics and Child Health, Paediatric Haematology-Oncology, Charlotte Maxeke Johannesburg Academic Hospital, Wits Donald Gordon Medical Centre, University of the Witwatersrand, Johannesburg, South Africa; ^2^ Computer Science, St Andrew's University, St Andrew's, United Kingdom; ^3^ Universitas Hospital, Bloemfontein, South Africa; ^4^ Paediatric Haematology-Oncology, University of the Free State, Bloemfontein, South Africa; ^5^ Paediatric Haematology-Oncology, University of KwaZulu-Natal, Durban, South Africa; ^6^ Greys Hospital, Pietermaritzburg, South Africa; ^7^ Faculty of Medicine and Health Sciences, Stellenbosch University and Tygerberg Hospital, Cape Town, South Africa; ^8^ Red Cross War Memorial Children’s Hospital, Cape Town, South Africa; ^9^ University of Cape Town, Cape Town, South Africa; ^10^ Department of Radiation Sciences, Paediatric Radiation Oncology, Charlotte Maxeke Johannesburg Academic Hospital, Wits Donald Gordon Medical Centre, University of the Witwatersrand, Johannesburg, South Africa; ^11^ Frere Hospital, East London, South Africa; ^12^ Paediatric Haematology-Oncology, Walter Sisulu University, East London, South Africa; ^13^ Nuclear Medicine, Center of Molecular Imaging and Theranostics, Johannesburg, South Africa; ^14^ Inkosi Albert Luthuli Central Hospital, Durban, South Africa; ^15^ Netcare Alberton Hospital, Alberton, South Africa; ^16^ Paediatric Haematology-Oncology, Steve Biko Academic Hospital, University of Pretoria, Pretoria, South Africa; ^17^ Paediatric Haematology-Oncology, Robert Mangaliso Sobukwe Hospital, Kimberley, South Africa; ^18^ Dr George Mukhari Hospital, Garankuwa, South Africa; ^19^ Paediatric Haematology-Oncology, Sefako Makgatho University, Garankuwa, South Africa; ^20^ Polokwane-Mankweng Hospital Complex, Polokwane, South Africa; ^21^ Paediatric Haematology-Oncology, University of Limpopo, Polokwane, South Africa; ^22^ Southern Africa Labour and Development Research Unit, School of Economics, Cape Town, South Africa; ^23^ Paediatric Haematology-Oncology, Chris Hani Baragwanath Academic Hospital, Wits Donald Gordon Medical Centre, University of the Witwatersrand, Johannesburg, South Africa; ^24^ School of Clinical Medicine, University of the Witwatersrand, Johannesburg, South Africa; ^25^ Pediatric, Medicins Sans Frontières, Geneva, Switzerland

## Abstract

**PURPOSE:**

Response assessment of classical Hodgkin lymphoma (cHL) with positron emission tomography-computerized tomography (PET-CT) is standard of care in well-resourced settings but unavailable in most African countries. We aimed to investigate correlations between changes in PET-CT findings at interim analysis with changes in blood test results in pediatric patients with cHL in 17 South African centers.

**METHODS:**

Changes in ferritin, lactate dehydrogenase (LDH), erythrocyte sedimentation rate (ESR), albumin, total white cell count (TWC), absolute lymphocyte count (ALC), and absolute eosinophil count were compared with PET-CT Deauville scores (DS) after two cycles of doxorubicin, bleomycin, vinblastine, and dacarbazine in 84 pediatric patients with cHL. DS 1-3 denoted rapid early response (RER) while DS 4-5 denoted slow early response (SER). Missing values were imputed using the k-nearest neighbor algorithm. Baseline and follow-up blood test values were combined into a single difference variable. Data were split into training and testing sets for analysis using Python scikit-learn 1.2.2 with logistic regression, random forests, naïve Bayes, and support vector machine classifiers.

**RESULTS:**

Random forest analysis achieved the best validated test accuracy of 73% when predicting RER or SER from blood samples. When applied to the full data set, the optimal model had a predictive accuracy of 80% and a receiver operating characteristic AUC of 89%. The most predictive variable was the differences in ALC, contributing 21% to the model. Differences in ferritin, LDH, and TWC contributed 15%-16%. Differences in ESR, hemoglobin, and albumin contributed 11%-12%.

**CONCLUSION:**

Changes in low-cost, widely available blood tests may predict chemosensitivity for pediatric cHL without access to PET-CT, identifying patients who may not require radiotherapy. Changes in these nonspecific blood tests should be assessed in combination with clinical findings and available imaging to avoid undertreatment.

## Teaser

Low-cost blood tests can predict chemosensitivity in pediatric HL, possibly reducing the need for radiotherapy.

## INTRODUCTION

Pediatric Hodgkin lymphoma, a focus cancer in the WHO Global Initiative for Childhood Cancer, is highly curable with traditional chemotherapy.^1^ Response-adapted management of classical Hodgkin lymphoma (cHL) using positron emission tomography-computerized tomography (PET-CT) has become the standard approach to determine chemosensitivity by monitoring functional and anatomic treatment response.^2,3^ Interim PET-CT (iPET-CT) assessment is considered to have excellent negative predictive value (NPV), but suboptimal positive predictive value (PPV), with a resolution of metabolic activity providing reassurance of adequate response, but limited confidence if the iPET-CT shows active disease.^4^ Response evaluation performed after two cycles of chemotherapy is used to identify patients who require treatment intensification with radiotherapy. Although PET-CT facilities are present at most major treatment centers in South Africa,^5^ there are periods when this imaging is not available. PET-CT facilities are also unavailable in most other African countries and many other low- and middle-income countries (LMICs),^6^ necessitating the search for low-cost alternatives.

**CONTEXT**

**Key Objective**
Using machine learning models, we predicted interim positron emission tomography-computerized tomography by using changes in blood test results in pediatric patients with classical Hodgkin lymphoma in 17 South African centers.
**Knowledge Generated**
The most predictive variable was the difference in absolute lymphocyte count, followed by changes in ferritin, lactate dehydrogenase, and total white cell count. Differences in erythrocyte sedimentation rate, hemoglobin, and albumin contributed less to the model.
**Relevance**
The findings are not currently applicable in clinical contexts. Once we have accrued more data, we will be able to run simulations to determine whether these findings can be used to direct treatment of individual patients.

The ability to predict which patients will survive (overall survival [OS]) or experience relapse or refractory disease (progression-free survival [PFS]) is well described, using disease-specific parameters such as bulky disease, B symptoms and tumor volume, as well as various individual blood tests.^7-9^ These include total white cell count (TWC), absolute lymphocyte count (ALC), absolute eosinophil count (AEC), erythrocyte sedimentation rate (ESR), lactate dehydrogenase (LDH), ferritin, copper, and albumin levels.^7,9-12^

Such parameters have been used to predict OS but not early response to chemotherapy. In South Africa, these blood tests are widely available and the cost of these tests in combination is significantly less than that of a single PET-CT scan. Despite satisfactory sensitivity, the diagnostic utility of these markers is limited by their lack of specificity, and the combination has not been studied in children and adolescents to predict treatment response.

Predictive modeling is an appealing option to aid early identification of patients with suboptimal iPET-CT response. We aimed to assess whether alterations in multiple blood markers, individually and collectively, could forecast rapid early response (RER) or slow early response (SER) on iPET-CT after two cycles of initial doxorubicin, bleomycin, vinblastine, and dacarbazine (ABVD) therapy through the application of machine learning models.

## METHODS

The study was approved by the University of the Witwatersrand Human Research Ethics Committee (M1711100), and the ethics committees of each participating pediatric oncology unit (POU), and registered on the National Health Research Database and the seven Provincial Health Research Databases in which POUs were located. Patients and guardians signed informed assent or consent for inclusion of data in this study.

Seventeen POUs prospectively enrolled patients younger than 19 years newly diagnosed with cHL onto a risk-stratified, response-adapted treatment protocol (SACCSG-HL-2018) from July 2016 to July 2022. Patients were staged according to the Ann Arbor staging system and risk-classified. Low-risk disease included stages IA, IB, and IIA; intermediate-risk disease was defined as stages I and II with risk factors (bulky or extranodal disease) and stage IIIA; and high-risk disease included stage II with both bulky disease and extranodal disease, stage III with any risk factors (B symptoms, bulky disease or extranodal disease), and all stage IV disease.^13^

For the majority of patients treated in the state sector, blood tests were performed at National Health Laboratory Services, an integrated network of accredited laboratories.^14^ Patients treated in the private sector had blood tests performed at Lancet Laboratories and Ampath Laboratories, which are multiply accredited and considered reference laboratories for various African countries.^15,16^ Blood samples were withdrawn by qualified nurses, phlebotomists, and doctors, and each test was performed once, as standard of care was provided and resources are limited.

Patients with low- and intermediate-risk disease received four and six courses of ABVD, respectively. Patients with high-risk disease received two courses of ABVD, followed by four courses of cyclophosphamide, vincristine, prednisone, and dacarbazine. Those with SER on the basis of persistent metabolic activity and those with bulky mediastinal disease received consolidation radiotherapy.

Data were entered into a secure, online REDCap database^17^ at each POU and collated in a central database by the PI who managed the database and initiated meetings, both online and in person, to facilitate uniformity of data capture and quality control.

Sample size estimation by the Bland-Altman method determined that data sets from 41 patients were required to calculate the receiver operating characteristic (ROC) curves to detect or fail to detect a difference of 3.6% from the reported AUC at 80% power and 5% significance.^18,19^ We analyzed relative changes in hematologic values (TWC, ALC, AEC, and hemoglobin) and nonspecific markers (ferritin, LDH, ESR, albumin, and copper) in comparison with Deauville scores (DS) on iPET-CT assessment after two cycles of ABVD. The first time point was at diagnosis and the second was at early response evaluation, at the start of the third cycle of chemotherapy. RER was classified as DS 1-3, while SER was classified as DS 4-5.^20^ We included patients who had a PET-CT performed both at baseline and response evaluation. The iPET-CT was performed as close as possible before the third cycle of chemotherapy without delaying the next cycle. Marker values at baseline and response evaluation were reported as mean, median, and range, and differences between clinical characteristics were assessed with the paired *t*-test for normally distributed data or the Wilcoxon signed-rank test for nonparametric data.^21,22^

We excluded patients with HIV infection as values of ESR, albumin, LDH, ferritin, TLC, AEC, and hemoglobin may differ from those of HIV-negative patients^23^ and may not reflect the same PPV for the iPET-CT response.

Abandonment and loss to follow-up were censored in the survival curves. OS was defined as the time from diagnosis to death from any cause. PFS was defined as the time from diagnosis to date of confirmation of relapsed or refractory disease, or death. Patients who did not experience an event were censored at the date of the last follow-up, and data lock was on 30 June 2023.

Data analysis was performed by health data science experts with Python 3.10 and scikit-learn 1.2.2. The baseline and interim blood test values were combined into a single difference variable. Data were split into training and testing sets for analysis with a set of machine learning models (logistic regression, random forests, naïve Bayes, and support vector machine classifiers). Missing values were imputed using a *k*-nearest neighbor (KNN) algorithm. For a given missing feature value in a row, KNN identifies *k* closest rows (neighbors) on the basis of similarity across other available features. These *k* closest rows are then used to determine the value of missing feature using summary statistics, such as mean and median. This provides an estimated value to impute for the missing data point. In our case, we used *k* = 2. We excluded copper from the final model because it had missing values due to unanticipated difficulties performing the test (37% missing at first presentation and 57% missing at interim assessment).

We derived random forest models that (1) predict response to chemotherapy and (2) supply important information into which characteristics contribute most to the response. Random forest is a machine learning algorithm comprising multiple decision trees to aggregate predictions from these trees and select the majority vote to determine the final outcome. Instead of using all available features, random selection is made at each split to reduce overfitting. We tested multiple standard machine learning classifiers and random forest achieved the highest performance on our data set, using methodology similar to Meti et al.^24^

ROC analysis was performed to assess the utility of baseline markers at diagnosis and at response evaluation for predicting iPET-CT response and to determine the best threshold values. For the ROC-derived AUC, *P* values were calculated to determine the cutoff points for the most accurate response-to-therapy prediction. Sensitivity, specificity, accuracy, PPV, and NPV were calculated using ROC curve threshold values. PPV was defined as the ability to predict SER on iPET-CT. Misclassification rates, calculated from the confusion matrix, express how often the confusion matrix is incorrect in predicting actual positive and negative outputs.

## RESULTS

### Patient Population

**FIG 1:**
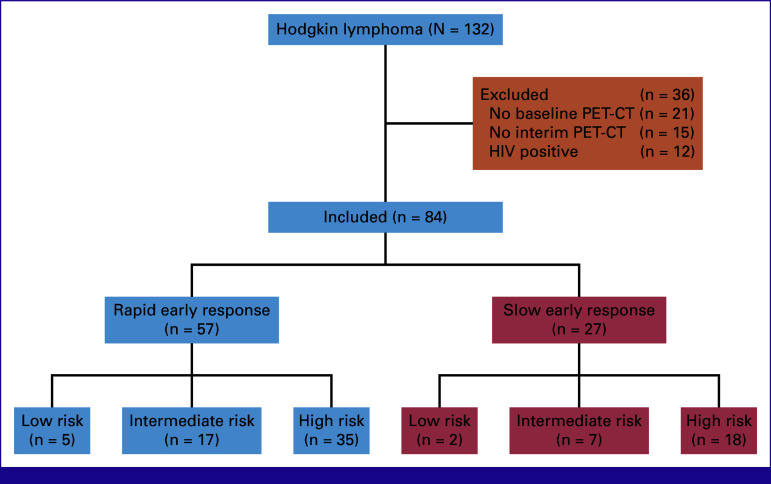
Interim assessment and risk stratification of pediatric patients on SACCSG-HL-2018. PET-CT, positron emission tomography-computerized tomography.

We enrolled 132 consecutive previously untreated patients with histologically proven cHL in the SACCSG HL-2018 study. PET-CT was performed at baseline in 111; of these, 96 had an iPET-CT performed. Twelve patients (13%) with HIV were excluded while 84 (87%) were HIV-negative and were analyzed in this study (Fig 1).

The median age was 9.7 years (range, 2.3-16.9 years; IQR, 6.9-12.7 years), with seven patients (8%) classified as low-risk, 24 (29%) as intermediate-risk, and 53 (63%) as high-risk. Comorbidities included tuberculosis in two patients, vanishing bile duct syndrome (1), idiopathic central precocious puberty (1), noncommunicating hydrocephalus (1), dilated cardiomyopathy (1), vitiligo (1), and decreased cardiac ejection fraction (1). After iPET-CT, 57 patients (68%) demonstrated RER and 27 (32%) demonstrated SER (Table 1).

### Hematologic values and nonspecific markers

**FIG 2:**
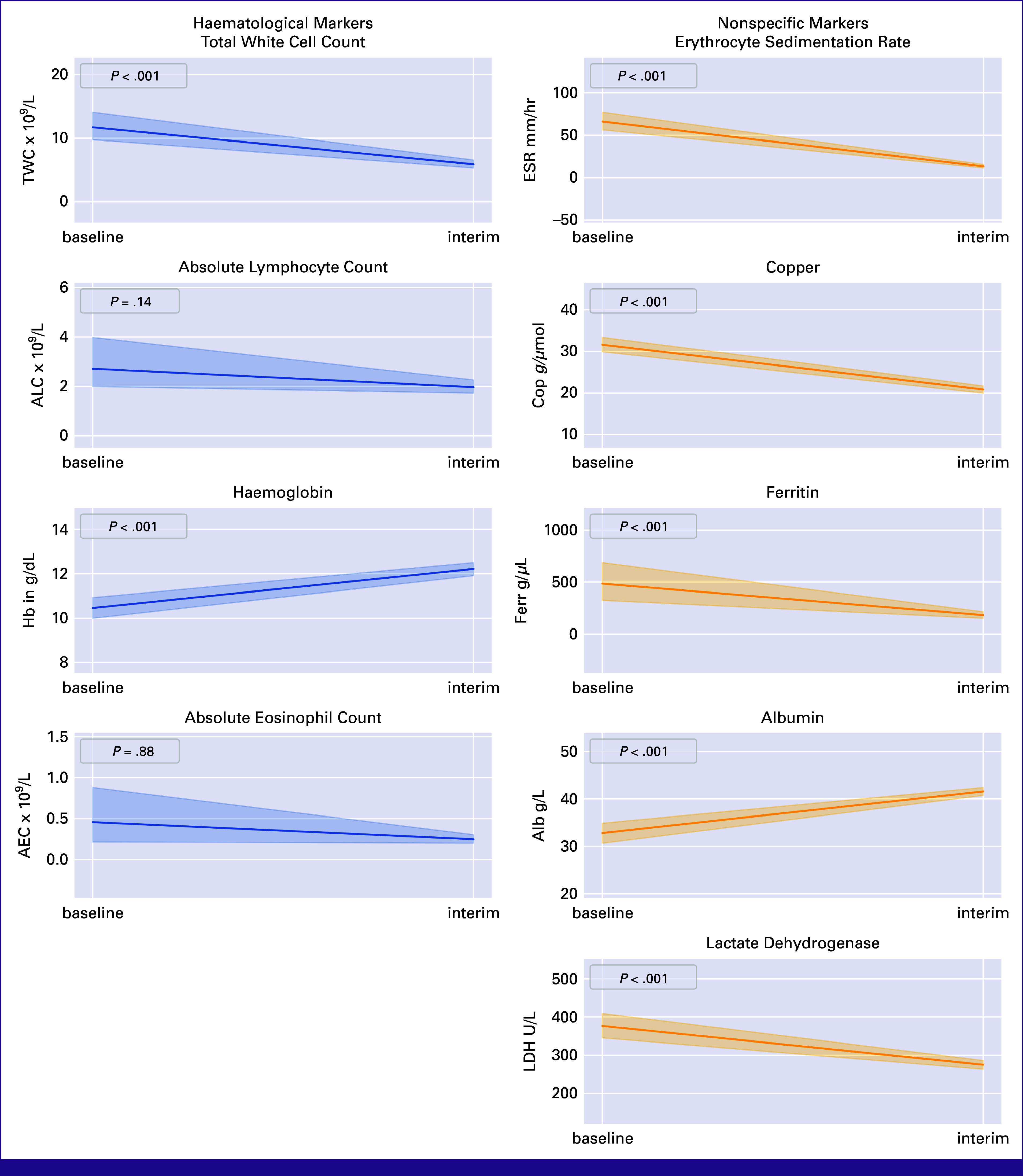
Hematologic parameters and nonspecific markers of pediatric patients on SACCSG-HL-2018 at baseline and interim analysis: (A) TWC, (B) ALC, (C) hemoglobin, (D) AEC, (E) ESR, (F) copper, (G) ferritin, (H) albumin, and (I) LDH. AEC, absolute eosinophil count; ALC, absolute lymphocyte count; ESR, erythrocyte sedimentation rate; LDH, lactate dehydrogenase; TWC, total white cell count.

None of the patients with hypoalbuminemia had nephrotic syndrome. At response evaluation, significant rises in hemoglobin and albumin (*P* < .001) and decreases in TWC, ESR, copper, LDH, and ferritin (all *P* < .001) were noted. Elevations in ALC and AEC were not statistically significant (*P* = .14 and .88, respectively; Fig 2; Data Supplement, Table S1).

**TABLE 1:** Baseline Characteristics of Children and Adolescents With Hodgkin Lymphoma

Parameter	No. (%)
Sex	
Male	21 (25)
Female	63 (75)
Age, years	
Median (IQR)	9.7 (6.9-12.7)
Range	2.3-16.9
BMI	
Obese/overweight	3 (4)
Adequate	63 (75)
<2 standard deviations	18 (21)
Histologic subtype	
Nodular sclerosing	51 (61)
Mixed cellularity	19 (23)
Lymphocyte-rich	2 (2)
Lymphocyte-depleted	1 (1)
HL NOS	11 (13)
Stage	
I	2 (2)
II	29 (35)
III	27 (32)
IV	26 (31)
B symptoms	
Yes	52 (62)
No	32 (38)
Bulky disease	
Yes	52 (62)
No	32 (38)
Autoimmune manifestations of HL	
Yes	7 (8)
No	77 (92)
Risk group	
Low risk	7 (8)
Intermediate risk	24 (29)
High risk	53 (63)
Response	
Rapid early response	57 (68)
Slow early response	27 (32)

Abbreviations: HL, Hodgkin lymphoma; NOS, not otherwise specified.

**FIG 3:**
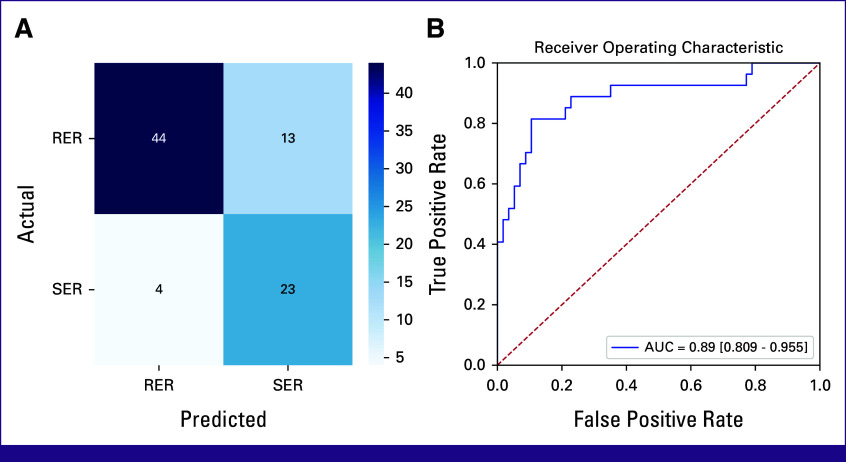
Prediction of chemosensitivity of pediatric patients with classical Hodgkin lymphoma. (A) Receiver operating characteristic curve. (B) Confusion matrix. RER, rapid early response; SER, slow early response.

After Bayesian optimization of model hyperparameters, random forest analysis achieved a validated test accuracy of 77% when predicting RER or SER from blood samples. The performance of other classifiers was inferior. The optimal model had a predictive accuracy of 86% and an ROC AUC of 93%, with a 95% confidence interval of 87%-98% (Fig 3A).

The sensitivity was 85% and the specificity was 86%, with a misclassification rate of 14% (Fig 3B). The model has a PPV of 74% in determining SER, and an NPV of 93% in predicting RER. Differences in ferritin, LDH, and TWC contributed 16%, 15%, and 14% respectively. Differences in ESR, hemoglobin, and albumin each contributed 11%-12%.

**FIG 4:**
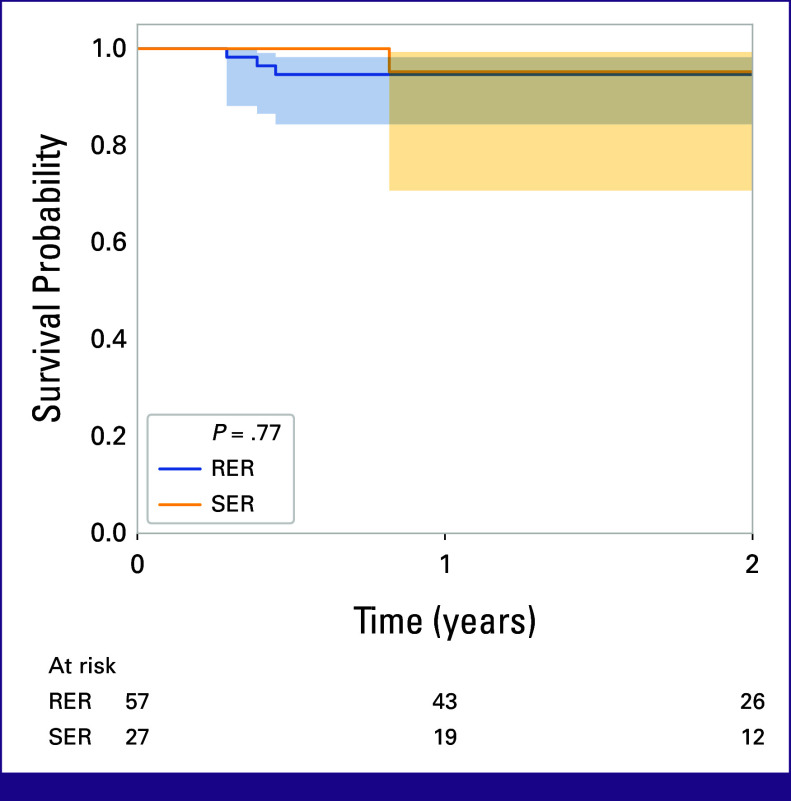
Kaplan-Meier curve of progression-free survival comparing patients with RER and SER. RER, rapid early response; SER, slow early response.

After a median follow-up of 2 years (range, 0.1-7.6 years), the 2-year OS was 95.3% for patients with RER and 96.4% for patients with SER (*P* = .72). PFS was 91.5% for patients with RER and 85.1% for patients with SER (*P* = .3; Fig 4).

## DISCUSSION

We show that the combined changes in certain hematologic parameters and nonspecific laboratory markers correlate with chemotherapy response on the basis of iPET-CT after two cycles of ABVD in pediatric patients with cHL. The most significant contributors to the study model were changes in ALC. Following closely were differences in LDH, AEC, and ferritin, which also showed strong predictive capabilities. Changes in TWC, ESR, hemoglobin, and albumin had a slightly lesser impact on the model's predictions. In combination, the changes in these simple blood markers offer more information than singly and can be used to predict response on iPET-CT.

As PET-CT has been shown to have a high number of false-positive FDG-avid lesions, it has more utility in excluding treatment failure and less in identifying treatment failure.^25,26^ The findings of this study highlight the potential benefits of monitoring changes in these blood tests as a means to predict the response to chemotherapy. Specifically, the observed changes in blood tests demonstrate a notable advantage in predicting RER rather than SER.

Although the changes in these blood tests cannot assist with the assessment of anatomic response, they offer value in assessing functional or metabolic response. In a previous study, an analysis of the individual baseline blood tests did not demonstrate the ability to predict 2-year OS.^13^ The current study does not focus on the prediction of survival but on the prediction of iPET-CT response after two cycles of ABVD.

Chemosensitivity is commonly predicted by iPET-CT performed after two cycles of chemotherapy, including ABVD.^27^ This response is used to guide further treatment by increasing treatment intensity, consolidating with radiotherapy or decreasing treatment intensity as in the de-escalation of escalated BEACOPP.^8,25,28^ iPET-CT has better PPV for predicting which patients will be long-term survivors but is less accurate at predicting which patients will develop progressive or relapsed disease.^25^ It thus has limited value in identifying patients who require intensification.

The limitations of nonspecific tumor markers in pediatric cHL highlight the need for more specific and sensitive diagnostic tools. PET-CT also has several limitations, including exposure to radiation, time commitment, and high cost, making it inaccessible in many low- and middle-income settings.^6^ Incorporating changes in sizes of malignant masses using chest x-rays and CT scans in combination with changes in blood tests may prove to be useful in resource-constrained settings.

There is thus a need to identify alternative methods that provide similar or better response assessment capabilities. By identifying biomarkers, clinicians could effectively monitor the progress of the disease and adjust the treatment regimen accordingly, leading to improved outcomes. Such markers must be at least as specific and sensitive as FDG-PET-CT imaging which, in itself, is insufficiently specific.

The ideal biomarker would have high PPV and NPV, be cost-effective, minimally invasive, readily available, sensitive and specific to changes in disease status with high PPV and NPV, and have a short turnaround time to facilitate decision making.^29^ Thymus and activation-regulated chemokine (TARC) is a promising biomarker that is easily measurable, sensitive, and specific for the disease, and may have a higher PPV than iPET-CT for progressive disease.^30,31^ The platform to set up a TARC testing system is less expensive than setting up a nuclear medicine facility, and an additional blood test is less invasive and inconvenient for patients.^29^ Other potential serum biomarkers are microRNAs associated with tumor-secreted extracellular vesicles in the circulation of patients with cHL.^32^ The combination of microRNA with TARC measurements has an AUC of 93%, 93.5% sensitivity, 85% specificity, and an NPV of 96%, but these tests are generally available only in the research setting, and certainly not in LMIC.

Most countries in Africa do not have access to PET-CT and, in those settings where PET-CT machines are available, access may still be limited because of the overwhelming patient numbers, cost, and transport limitations.^6^ Access to traditional chemotherapy is still limited in many countries.^33,34^ Salvage options such as autologous stem-cell transplant and novel targeted agents such as drug-conjugated antibodies and immune checkpoint inhibitors are out of reach for the majority.^35^ Nevertheless, relatively high survival rates have been achieved with conventional chemotherapy and radiotherapy in many countries using a strategic approach^34,36^ and the results of this study may contribute to improving response-adapted treatment in similar resource-constrained settings.

The ability to predict RER is clinically significant, enabling health care professionals to make informed decisions about treatment plans and allowing for timely adjustments if necessary. The choice to avoid radiotherapy has long-term consequences, decreasing the chances of the development of subsequent malignant neoplasms and other late effects of radiotherapy.^3^ Conversely, patients who demonstrate SER may require alternative treatment strategies or modifications to their current regimen. By identifying SER timeously, health care providers can proactively address the situation, potentially avoiding delays in achieving the desired therapeutic outcome.

In this study, we used advanced machine learning techniques applied to baseline and interim data to derive a reliable method for discrimination between later RER and SER. A range of classification models, each having strong previous evidence of utility and performance, were fine-tuned using Bayesian optimization to identify the model setting that gave the best validated model performance. The model with the best performance was random forests, a nonparametric stochastic ensemble method that aggregates the results of many individual decision tree models to both guard against overfitting the supplied data and return a model with low predicted error when applied to new and/or unseen cases. The random forest approach method also allows the identification of the most important factors that underpin a prediction of later RER or SER. We show that differences in ESR, ferritin, and albumin are less important for this study, suggesting that future studies should prioritize differences in hemoglobin, ALC, LDH, AEC, and TWC.

More recently, an important goal has been to predict the subset of patients who will relapse to intensify treatment for these patients.^29^ iPET-CT alone is insufficient for this purpose, but the combination of blood tests and iPET-CT results may in time yield further useful information. The potential correlation between iPET-CT and patient survival is as yet inconclusive in our patient cohort, as demonstrated by the similar 2-year OS in patients with RER and SER. We may postulate that by escalating treatment guided by iPET-CT, the survival disparity has effectively been reduced. The quest to predict relapse or progression continues, especially in settings where salvage options are limited.

Although the results of this study are useful in predicting iPET-CT response, the challenge remains to tailor treatment safely and appropriately, to effect cure with minimal side effects, and to identify those patients who require treatment intensification with accuracy. These findings highlight the importance of monitoring changes in blood tests as they offer valuable insights into the prediction of RER to chemotherapy. By leveraging these predictive markers, health care professionals in resource-limited settings may be able to optimize treatment plans, personalize care, and enhance patient outcomes. However, caution should be exercised in settings with potentially diverse patient populations: different patient groups may exhibit variations in baseline hematologic parameters or levels of nonspecific markers. These variations may be influenced by a range of factors, including underlying medical conditions, demographic characteristics, and environmental factors.

Challenges now include the translation of these results into a format with clinical utility. The envisaged solution is the development of a practical scoring system or a mobile smartphone application. Such tools would allow for the input of blood test results to generate a score indicating whether radiotherapy can be safely avoided. In settings where radiation therapy is not available, such tools may not be as relevant, but may still be of value to predict survival. Ultimately, the goal is to tailor the treatment regimen for each patient to achieve maximal efficacy with minimal side effects while accurately identifying patients who require treatment intensification.

The study required a minimum of 41 complete patient data sets to ensure precise evaluation through ROC curves. Because of the unavailability of complete data sets, imputation techniques were used. The limitation associated with imputing missing values is anticipated to be addressed to some extent by using 84 data sets, double the required sample size calculation. The final random forest model selected would benefit from more baseline, interim, and outcome data, and hence the results reported in this study are indicative of future model performance rather than definitive. The scope of this investigation solely encompassed analysis of the potential of changes in blood tests to predict iPET-CT response, without considering the subsequent relapse status of these patients. We did not include risk classification at presentation in the machine learning models as the sample size was too small to provide suitable power.

In conclusion, the pooled changes in certain hematologic parameters and nonspecific laboratory markers exhibit a strong correlation with chemosensitivity, as evaluated through iPET-CT scans after two cycles of ABVD treatment in pediatric patients with cHL. We predicted RER or SER from blood samples with a high level of accuracy. In resource-constrained environments with limited access to PET-CT, these findings offer potential tools to predict chemosensitivity. In such settings, these changes could serve as substitutes for iPET-CT and thus assist with the identification of pediatric patients with cHL who can safely forgo radiotherapy and avoid its subsequent late effects. These results are not definitive in isolation and should be interpreted in conjunction with other diagnostic tests and clinical findings to establish chemosensitivity accurately. Although these hematologic and nonspecific tumor markers provide valuable insights into disease progression and treatment response, their lower specificity imposes limitations. Consequently, additional research efforts are warranted to identify more specific response-assessment tools for pediatric cHL to give patients in LMIC the best possible chance of cure with high quality of life.

## Data Availability

The dataset for this study is available on request.

## APPENDIX 1. LIST OF SOUTH AFRICAN CHILDREN'S CANCER STUDY GROUP MEMBERS WITH E-MAILS

David Stones (StonesDK@ufs.ac.za); Ruellyn Cockroft (rcockcroft@xtra.co.nz); Alan Davidson (alan.davidson@uct.ac.za); Anabela Andrade (a_andrade@mweb.co.za); Ane Buchner (ane.buchner@up.ac.za); Ann Van Eyssen (ann.vaneyssen@uct.ac.za, annveyssen@tiscali.co.za); Barry van Emmenes (barry.vanemmenes@gmail.com); (bernard.goodwin@wits.ac.za); Biance Rowe (biance_r@hotmail.com); Clare Stannard (Clare.Stannard@uct.ac.za); Cristina Stefan(cristinastefan10@gmail.com); David Reynders (David.Reynders@up.ac.za); Diane MacKinnon (dianemacleask@gmail.com); Elmarie Mathews (mathews.elmarie@yahoo.co.uk); Farieda Desai (farieda.desai@gmail.com); Gesami Steytler (gesamisteytler@gmail.com); Gita Naidu (gita.naidu@wits.ac.za); Janet Poole (Janet.Poole@wits.ac.za); Jeanette Parkes (jeanette.parkes@uct.ac.za); Johani Vermeulen (johani.vermeulen@gmail.com); Karin Lecuona (Karin.Lecuona@uct.ac.za); Karla Thomas (karlamthomas@yahoo.com, Karla.Thomas@echealth.gov.za); Kate Bennett (kate_bennett@hotmail.com); Kershinee Reddy (kershinee@yahoo.com); Keshnie Moodley (drkeshnie@gmail.com); Komala Pillay (Komala.Pillay@uct.ac.za); Mariana Kruger (marianakruger@sun.ac.za); Jaques van Heerden (jaquesvanheerden@gmail.com); Linda Wainwright (sjaspan@iafrica.com); Leila Schoonraad (Leilaschoonraad@gmail.com); Lourens de Jager (ldejager@icon.co.za); Mairi Bassingthwaighte (mairi.bass@gmail.com); Manickavallie Vaithilingum (vaithil@medis.co.za); Nicolene Moonsamy (nicolenemoonsamy@gmail.com); Oloko Wedi (olokowedi@yahoo.com); Palessa Radebe (palessa.radebe@gmail.com); Ronelle Uys (ruys@sun.ac.za); Stelios Poyiadjis (stelios.poyiadjis@wits.ac.za); Rajendra Thejpal (thejpal@ukzn.ac.za); Rosemarie Schwyzer (Rosemarie.Schwyzer@wits.ac.za); Johannes Du Plessis (duPlesJP@ufs.ac.za); Candice Hendricks (candice_hendricks@outlook.com); Pieter Hesseling (pbh@sun.ac.za); Barry Vanemmenes (Barry.Vanemmenes@echealth.gov.za); Miss Judy Schoeman (Judy.schoeman@up.ac.za); Mohamed Adamjee (dradamjee@yahoo.co.za); Milind Chitnis (chitnis.m@gmail.com); Pat Hartley (phartley@uct.ac.za); Wendy Mathiassen (wrwm@iafrica.com); Alistair Millar(Alistair.millar@uct.ac.za); Sr Colleen Wright (cawr@sun.ac.za); Nadia Beringer (nadia.beringer@gmail.com); Ngoakoana Mahlachana (ngoakoanam@yahoo.com); Thandeka Nkabi (thandekankabi@yahoo.com); Jade Flood (jade@floodgates.co.za); Mampoi Jonas (mmorienyane@yahoo.com); Hamidah Van Staaden (hbvanstaaden@yahoo.com); Thurandrie Naicker (thuran.naiker@uct.ac.za); Pawel Schubert (pawels@sun.ac.za); Bulelwa Masoka (bulelwa.masoka@yahoo.com)
